# Predicting dyslipidemia incidence: unleashing machine learning algorithms on Lifestyle Promotion Project data

**DOI:** 10.1186/s12889-024-19261-8

**Published:** 2024-07-03

**Authors:** Senobar Naderian, Zeinab Nikniaz, Mahdieh Abbasalizad Farhangi, Leila Nikniaz, Taha Sama-Soltani, Parisa Rostami

**Affiliations:** 1 Department of Health Information Technology, School of Management and Medical Informatics, Tabriz University of Medical Sciences, Tabriz, Iran; 2 Student Research Committee, Tabriz University of Medical Sciences, Tabriz, Iran; 3 Liver and Gastrointestinal Diseases Research Center, Tabriz University of Medical Sciences, Tabriz, Iran; 4 Department of Community Nutrition, Faculty of Nutrition, Tabriz University of Medical Sciences, Tabriz, Iran; 5 Tabriz Health Services Management Research Center, Tabriz University of Medical Sciences, Tabriz, Iran; 6 Department of Health Information Technology, School of Management and Medical Informatics, Tabriz University of Medical Sciences, Tabriz, Iran

**Keywords:** Dyslipidemia, Machine learning, Predictive modeling, Lifestyle promotion project, Multi-layer perceptron neural network, Random forest, Data preprocessing, Feature selection

## Abstract

**Background:**

Dyslipidemia, characterized by variations in plasma lipid profiles, poses a global health threat linked to millions of deaths annually.

**Objectives:**

This study focuses on predicting dyslipidemia incidence using machine learning methods, addressing the crucial need for early identification and intervention.

**Methods:**

The dataset, derived from the Lifestyle Promotion Project (LPP) in East Azerbaijan Province, Iran, undergoes a comprehensive preprocessing, merging, and null handling process. Target selection involves five distinct dyslipidemia-related variables. Normalization techniques and three feature selection algorithms are applied to enhance predictive modeling.

**Result:**

The study results underscore the potential of different machine learning algorithms, specifically multi-layer perceptron neural network (MLP), in reaching higher performance metrics such as accuracy, F1 score, sensitivity and specificity, among other machine learning methods. Among other algorithms, Random Forest also showed remarkable accuracies and outperformed K-Nearest Neighbors (KNN) in metrics like precision, recall, and F1 score. The study’s emphasis on feature selection detected meaningful patterns among five target variables related to dyslipidemia, indicating fundamental shared unities among dyslipidemia-related factors. Features such as waist circumference, serum vitamin D, blood pressure, sex, age, diabetes, and physical activity related to dyslipidemia.

**Conclusion:**

These results cooperatively highlight the complex nature of dyslipidemia and its connections with numerous factors, strengthening the importance of applying machine learning methods to understand and predict its incidence precisely.

## Introduction

Dyslipidemia is described as changes in the plasma lipid profile which contains increased cholesterol, high low-density lipoprotein, elevated triglyceride, and low high-density lipoprotein [1]. It is connected to more than four million deaths every year all around the world [2]. These conditions can make human beings susceptible to some other diseases such as cardiovascular disease [1, 3], stroke [4], non-alcoholic fatty liver disease (NAFLD), and acute pancreatitis [5]. In 2016, Parray et al. declared that dyslipidemia was detected in 82.6% of men and 47.6% of women in the range of 5–9 years old and 24.7% of men and 35.9% of women in the range of 15–19 years old in Kashmir [6]. In 2019, Sadegh Tabrizi et al. showed that hypercholesterolemia, elevated LDL-C, hypertriglyceridemia, low HDL-C, and dyslipidemia was seen in 29.4%, 10.3%, 62.3%, 41.4%, and 83.3% of the population in urban and rural areas of the Northwest of Iran [7].

As dyslipidemia is one of the important risk factors for coronary artery disease, stroke [8], non-alcoholic fatty liver disease (NAFLD) [9], chronic kidney disease, diabetic nephropathy [10], preeclampsia [11], airflow obstruction [12], and dementia [13], it is crucial to predict its incidence both in people who are at risk but do not have this condition yet and people who already suffer from it. Screening and treatment of juveniles with dyslipidemia have eminent significance in decreasing cardiovascular disease in the future [14]. Paying attention to drugs, healthy nutrition, and proper lifestyles of people at risk of dyslipidemia is important [7].

Artificial Intelligence (AI) is useful in medicine in several regions including screening [15], disease diagnosis, drug development, and treatments [16]. Recently, the idea of using AI for analyzing data is one of the notable topics [17]. AI assists in managing and determining large datasets effectively with high accuracy [17, 18]. Feature extraction is another method that can give us a better comprehension of the data with a suitable prediction precision [17]. Machine Learning (ML) techniques were used for categorizing high risk patients for COVID-19 in addition to its diagnosis [18].

Ensemble learning is an approach in machine learning that aims to boost predictive accuracy by merging predictions from multiple models. This methodology looks for minimizing prediction errors that may happen due to overgeneralization [19]. By utilizing a varied set of models that operate individually, ensemble methods can efficiently moderate prediction errors. Fundamentally, the ensemble method aggregates the unique outputs to produce a combined prediction. Regardless of containing multiple basis models, the ensemble acts and provides outputs as if it were a single model [20, 21]. The fundamental point of ensemble models is to integrate multiple weak learners into robust learners, thereby enhancing overall model accuracy [22]. Common sources of inconsistencies between actual and predicted values in machine learning models include noise, variability, and bias [23]. Bagging, boosting, stacking, and voting, are among the notable approaches in this domain, offering improved predictive performance by combining the outputs of multiple base learners [24, 25]. One of the most frequently used ensembled algorithms is voting [22]. Voting classifiers combine predictions from individual models to improve accuracy and robustness [21, 25]. While ensemble methods have shown promise in various applications, including disease prediction and diagnosis, their specific role in predicting dyslipidemia requires further investigation.

Several recent studies have investigated the use of machine learning algorithms in predicting dyslipidemia and correlated factors.

Cui et al., demonstrated superior performance of long short-term memory (LSTM) method, a subset of deep learning, in predicting dyslipidemia among steel workers achieving accuracy exceeding 95% [26]. In contrast, traditional recurrent neural networks showed lower accuracy [26]. In France, researchers used boosted version of Logistic regression (LR), decision tree models and XGBoost to predict diabetes incidence, with accuracies ranging from 67 to 77% [27]. Marateb et al., applied various machine learning algorithms, including supported vector machines, decision trees, neural networks, and logistic regression, to predict dyslipidemia in children and juveniles, achieving an average accuracy and precision of 92% and 94% respectively. [2].

Gutiérrez-Esparza et al., analyzed a dataset of 2,621 participants to identify major factors associated with dyslipidemia, such as body mass index, age, and anxiety. The Random Forest algorithm showed the highest efficacy, with an 80% accuracy in predicting dyslipidemia risk [28]. Using deep learning techniques, Hyerim Kim et al., investigated the influence of nutritional intake on dyslipidemia, revealing moderate accuracy (0.58%) in dyslipidemia prediction among participants aged 40 to 69 years [29]. Tavolinejad et al., remarked that the random forest model ensemble model showed advanced predictive accuracy for hypertension care coverage, with an AUC going beyond 0.89 for all machine learning models. They stated that younger age, male sex, and being single/divorced were steadily related to a reduced probability of obtaining care [30].

Akyea et al., showed that ensemble learning outperformed basic machine learning algorithms in detection of familial hypercholesterolemia (FH). achieving AUC values beyond 0.89, compared to logistic regression with an AUC of 0.81 [31].

Ensemble learning has been employed across various domains, near or far from the medical domain, beyond these studies. Buyrukoğlu et al., demonstrated the superiority of machine learning models, particularly AdaBoost, in accurately predicting the population of Escherichia coli in agricultural ponds based on weather station measurements [32]. In a study focusing on early prediction of type 2 diabetes, Buyrukoğlu proposed a hybrid feature selection approach combining correlation matrix with heatmap and sequential forward selection, effectively identifying optimal features for diabetes detection, and outperforming other machine learning algorithms [33].

Despite these advancements, there is a lack of recent studies predicting dyslipidemia incidence in Iran using machine learning and ensemble learning methods. Hence, our study aims to fill this gap by predicting dyslipidemia incidence based on data from Lifestyle Promotion Project (LPP) using machine learning algorithms.

### Research questions

- How do different machine learning algorithms, including ensemble models, perform in predicting dyslipidemia incidence?
- What are the key factors associated with dyslipidemia according to the machine learning models?

## Methods

To ensure the proposed method effectively addresses the research problems identified, we adopted a comprehensive approach integrating machine learning techniques with lifestyle promotion project dataset. The LPP, a longitudinal community-based initiative aimed at preventing and controlling non-communicable diseases (NCDs) in East Azerbaijan Province, Iran. The design had two important parts; stage I was a cross-sectional prevalence study of NCDs and their associated risk factors which was accomplished from Feb 2014 to Apr 2014. Stage II was a prospective follow-up study initiated in Feb 2016 [34]. In phase I, 3000 patients (15–65 years) who were 1500 households (150 clusters) living in East Azerbaijan province were selected inadvertently based on postal code from six cities in this province. You can find comprehensive details in the study protocol [35]. The study explores the use of LPP in Iran to prevent non-communicable diseases in developing countries, focusing on discrepancies in NCD frequency and results before and after lifestyle interventions [34].

### Dataset

The LPP study dataset was used to collect information on risk factors according to WHO format, including socio-demographic, Angina, smoking, physical activity, anxiety, diet, food security, food safety, biochemical measurements, daily intakes, biomedical parameters, and lifestyle promotion interventions.

### Preprocess and merging

The study involved collecting data from four separate sources, each with unique columns. Merging the data required a comprehensive analysis, rather than using ***pandas.merge***. Two sources contained biomedical assessment data, while the remaining files contained physical examinations, questionnaire responses, and missing data.

1. The code read two CSV files into separate ***dfs***, df1 and df2, and specified columns using commands like ***set = set(df.columns), diff = sorted(set1 - set2).*** It then copied columns, reorders df2 according to df1, and iterated over columns to check if they exist.
2. The second step involved combining data from two biomedical sources, questionnaires, and physical assessments to create an integrated data set for further analysis.
3. According to our inclusion criteria, patients with at least one of the biomedical test results: Fast Blood Sugar, Ferritin, Anemia, Alanine transaminase, Cholesterol, High-Density Lipoprotein, Hemoglobin, Aspartate aminotransferase, Serum Vitamin D status, were included. The pandas library was used to create a ***df*** of data, resulting in a csv file with 548 columns and 8814 rows.

### Null handling

We used Python packages ***numpy*** and ***pandas*** to handle null and missing data in the dataset. We converted columns to numeric values and identified missing or empty columns and remove them from the dataset. We also assigned numerical values to empty cells to represent features, according to the team’s expert opinion. The final dataset consisted of 502 columns for patients and 132 rows for features and targets.

### Target selection

To achieve the study purpose, our team’s expert selected 5 distinct target columns representing or related to dyslipidemia: “Dyslipidemia” and “HDL category” which were categorized in 2 classes (presence or absence / low or high). “Cholesterol category” which was categorized in 3 classes indicating the order of blood cholesterol levels from 1 (the lowest) to 3 (the highest). Similarly “Triglyceride rating” and “LDL rating” which were categorized in 4 classes representing Triglyceride and LDL cholesterol levels from 1 (the lowest) to 4 (the highest).

### Normalization

The dataset was scaled using three normalization methods: StandardScaler, min-max, and robust normalization. StandardScaler transforms the data to a mean and standard deviation of 1, while MinMaxScaler, rescales it in a specific range. RobustScaler rescales the data by subtracting the first quartile but is less precise for detecting outlier data. The dataset was converted to a numeric format, and targets were extracted. Three scaler ***dfs*** were instantiated, fitted to the data using their respective ***fit_transform*** methods, and target columns were added to each scaled ***df***.

### Feature selection

We applied 3 different feature selection algorithms to Select 10 best objects associated with each target variable. chi-square, mutual information, and ANOVA F-value were used to analyze feature importance. Inspired by previous studies on feature selection techniques in medical data analysis, we adopted the Chi-Square method, mutual information-based feature selection, and an approach utilizing mutual information theory. Sikri et al., demonstrated the importance of pre-processing data to fulfill the assumptions of the Chi-Square method, highlighting its impact on feature ranking [36]. Sulaiman and Labadin, proposed a feature selection method based on mutual information criterion, showcasing its effectiveness in improving machine learning model performance [37]. Additionally, Hoque et al., introduced a greedy feature selection method using mutual information theory, which demonstrated high classification accuracy across multiple datasets [38]. These studies informed our selection of feature selection techniques and provided valuable insights into their application in medical data analysis. Three feature selection objects were created using these score functions, fitted to imputed data, and the top 10 features were selected using the ***SelectKBest*** class from the ***sklearn.feature_selection*** module.

**Fig. 1:**
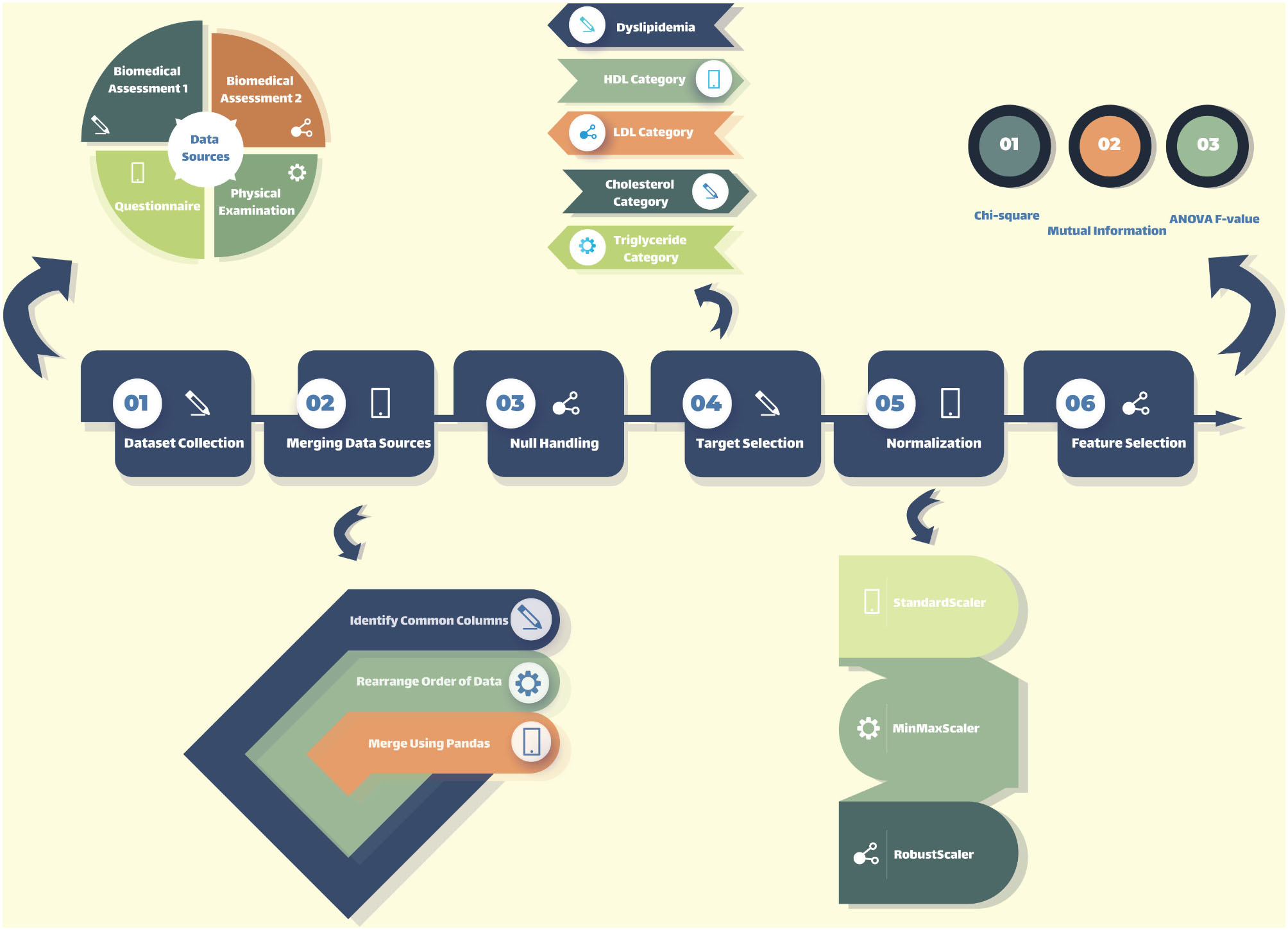
Unified methodology framework for effective classification and feature-outcome analysis

In Fig. 1, a visual representation of the comprehensive method is presented, illustrating the interplay between distinct steps and pathways within each, to achieve our goal.

### Machine learning methods

The dataset was classified using algorithms such as Decision Tree, K Nearest Neighbor, Naïve Bayes, Random Forrest, Support Vector Machine, and Neural Network. The selection of machine learning algorithms for dyslipidemia prediction was based on their strengths and characteristics. Decision Tree (DT) was chosen for its simplicity and interpretability, Random Forest (RF) for its robustness, Naïve Bayes for its computational efficiency, Neural Network (NN) for its ability to model complex patterns, K-Nearest Neighbors (KNN), Support Vector Machine (SVM), and Ensemble Learning through Voting [39–41].

Each algorithm involved setting a random seed, loading the dataset, extracting target columns, converting features, splitting data, calculating class weights, training the classifier model, evaluating performance using different metrics, and plotting results. Three feature selection methods were used for each target variable, followed by three normalization methods. Nine combinations of feature selection methods and normalization methods were applied to evaluate the performance of each algorithm for target variables. Metrics included accuracy, precision, recall, F1 score, and specificity. The study establishes a novel approach by analyzing the LPP study dataset using machine and deep learning techniques and investigating the optimized performance of each model through the intersection of normalization and feature selection methods.

In addition to the mentioned machine learning algorithms, an ensemble learning algorithm was trained for each target variable using the same normalization and feature selection methods. Through the process of ensemble learning method individual base classifiers, like Decision Tree and Random Forest, were trained on the preprocessed dataset using the same normalization and feature selection methods for consistency across the ensemble. The predictions of these base classifiers were then combined using either a “hard” voting scheme, where the majority vote determines the final prediction, or a “soft” voting scheme, where probabilities predicted by each base classifier are averaged. Subsequently, the ensemble classifiers underwent evaluation using cross-validation techniques to estimate their performance on unseen data. Accuracy and F1 score were calculated and compared with those of individual classifiers. Additionally, confusion matrices are generated to provide detailed analysis of the ensemble model’s performance.

For external validation, another study dataset titled “Effect of cranberry supplementation on liver enzymes and cardiometabolic risk factors in patients with NAFLD: a randomized clinical trial” was used [42]. This dataset included four out of five target variables of interest, including LDL, HDL, TG, and cholesterol categories. The accuracy of the trained models on this external dataset was also reported in the respective target variable tables.

**Fig. 2:**
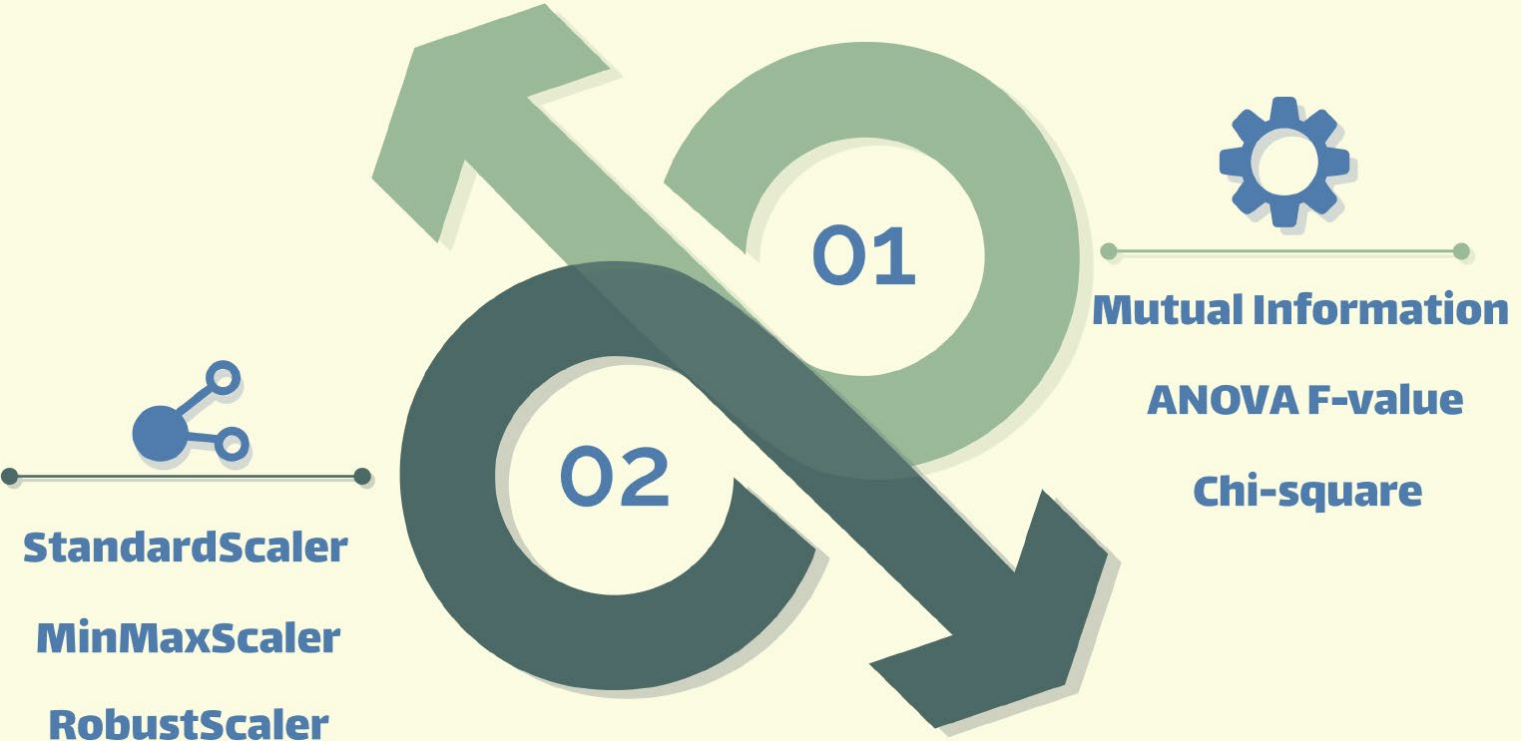
Novelty of the method

In Fig. 2, the novelty of method, which is an interaction between normalization and feature selection methods, is presented.

### Data availability

The codes used in this article, along with the dataset supporting our conclusions, are accessible via the GitHub repository linked here.

Additionally, it’s important to note that three versions of the dataset are available, each resulting from a different normalization method:

csv1: Result of Min-Max normalizer.

csv2: Result of StandardScaler normalizer.

csv3: Result of RobustScaler normalizer.

### Connection with research questions

### To address RQ 1 regarding the performance of machine learning algorithms, we applied a range of algorithms and ensemble learning techniques, aligning with the study’s overarching goal

RQ 2, concerning the identification of key factors associated with dyslipidemia, was addressed through feature selection methods, which identified the most influential features contributing to dyslipidemia risk prediction.

### Ethical approval and consent to participate

It is crucial to note that the study did not originally create a dataset, but rather utilized existing data from the Lifestyle Promotion Project (LPP) study. The LPP is a longitudinal community-based plan for the prevention and control of non-communicable diseases in East Azerbaijan Province, Iran [34]. The use of this data aligns with our commitment to ethical considerations and ensures the privacy and confidentiality of participants.

## Results

We sought to determine the best and optimized combination of feature selection and normalization methods which would result in the best performance of each model for 5 distinct targets: Dyslipidemia and HDL Category (2 classes), Cholesterol Category, Triglyceride rating and LDL rating (3, 4 and 4 classes, respectively). Below you can see the results that show the best feature sets for each algorithm and algorithm metrics.

### Dyslipidemia target

Table 1 provides information about the best combination of feature selection and normalization methods and the resulting feature sets for each algorithm. Decision Tree, Random Forest and Naïve Bayes showed a common feature set and Neural Network, Support vector Machine and KNN showed another. Also as shown in Table 1, Neural Network attained the highest accuracy of 0.98, surpassing in capturing complicated patterns and it stands out as the top performer for this target. Decision Tree and Random Forest also performed generally well, with accuracies of 0.84. Evidently Naïve Bayse could achieve this accuracy too, but it performed poorly in the matter of sensitivity that could represent the performance of this algorithm for assuming independence among features, which may not have been true in this context. This makes Naïve Bayse a bad option for detection of positive instances. KNN and SVM showed moderate accuracies, with KNN somewhat beating SVM (0.81 and 0.68 respectively.

**Table 1:** Feature selection, normalization and performance metrics for dyslipidemia target

Algorithm	Feature selection	Normalization	Obtained features from “feature selection” and “normalization” combination	Accuracy	Precision	Sensitivity	Specificity
Decision Tree	Chi2 score	MinMax	[‘Female Waist Circumference‘(cm), ‘SerumvitD‘(ng/ml), ‘SerumvitD’ (sufficient/deficient), ‘Metabolic syndrome‘(yes/no), ‘Total blood pressure‘(yes/no), ‘Copper‘(intake/mcg/day), ‘Chromium‘(intake/mcg/day), ‘Atocopherol‘(intake/mg/day), ‘Sugar‘(intake/g/day), ‘Vegetable oil‘(intake/g/day)]	0.80	0.99	1.0	0.99
Random Forest	Chi2 score	MinMax		0.84	0.99	0.98	1.0
Naïve Bayes	Chi2 score	MinMax		0.84	0.80	0.10	0.98
**Neural Network**	F score	STD	[‘Female Waist Circumference‘(cm), ‘History of Anxiety‘(yes/no), ‘SerumvitD‘(ng/ml), ‘SerumvitD‘(sufficient or deficient), ‘Metabolic syndrome‘(yes/no), ‘Carbohydrate‘(intake/g/day), ‘Chromium‘(intake/mcg/day), ‘VitaminD‘(intake/mcg/day), ‘Suger‘(intake/g/day), ‘Vegetable oil‘(intake/g/day)]	**0.97**	0.97	0.83	0.99
Support vector Machine	F score	STD		0.68	0.75	0.74	0.70
KNN	F score	Robust		0.81	0.99	0.98	1.0

### Cholesterol category target

Table 2 analyzed the performance of various algorithms, with Neural Network outperforming others by achieving an accuracy of 0.88. Decision Tree and Random Forest performed well in handling complex data relationships, with perfect precision, sensitivity, and specificity (1.0). KNN followed closely with an accuracy of 0.61 and moderate performance in precision, sensitivity, and specificity (0.89, 0.81, 0.91). Naïve Bayese achieved an accuracy of 0.61 but struggled in identifying certain classes (sensitivity of 0.38). SVM might have had challenges in precisely splitting data into separate classes (accuracy of 0.49). The models’ generalization to unseen data beyond the training set was consistent, with slight variations observed between the dataset and the external dataset.

**Table 2:** Feature selection, normalization and performance metrics for cholesterol category target

Algorithm	Feature selection	Normalization	Obtained features from “feature selection” and “normalization” combination	Accuracy	Precision	Sensitivity	Specificity	External dataset accuracy
Decision Tree	Mutual info	MinMax	[‘Age’, ‘History of diabetes‘(yes/no), ‘Angina grade’ (severe/not severe), ‘SerumvitD‘(ng/ml), ‘Physical activity‘(1 = inactive, 2 = minimally, 3 = highly active), ‘Prehypertension‘(yes/no), ‘CobalaminB12‘(intake/mcg/day), ‘Dietary fiber‘(intake/g/day), ‘Daily serving of grains intake‘(intake/g/day), ‘Daily serving of fats intake(intake/g/day)’]	0.61	1.0	1.0	1.0	0.56
Random Forest	Mutual info	STD and Robust	[‘Number of family members’, ‘Age’, ‘History of diabetes‘(yes/no), ‘Angina grade‘(severe/not severe), ‘serumvitD‘(ng/ml), ‘Physical activity‘(1 = inactive, 2 = minimally, 3 = highly active), ‘Prehypertension‘(yes/no), ‘CobalaminB12‘(intake/mcg/day), ‘Dietary fiber‘(intake/g/day), ‘Daily serving of grains intake‘(intake/g/day)]	0.67	1.0	1.0	1.0	0.69
KNN	Mutual info	STD and Robust		0.61	0.89	0.81	0.91	0.66
**Neural Network**	Mutual info	STD and Robust		**0.88**	0.99	1.0	1.0	**0.86**
Support vector Machine	Mutual info	STD and Robust	[‘Sex’, ‘Age’, ‘Level of education‘(illiterate/undergraduate/college), ‘Female Waist Circumference‘(cm), ‘Male Waist Circumference‘(cm), ‘Hypertension categorical(1 = normal, 2 = Pre-hypertension, 3 = normal with medicine, 4 = Pre-hypertension with medication, 5 = grade 1, 6 = grade 2), ‘Blood glucose level(normal/ prediabetic/ diabetic)’, ‘Metabolic syndrome‘(yes/no), ‘Waist to height ratio’, ‘Total blood pressure‘(yes/no)]	0.49	0.60	0.63	0.78	0.58
Naïve Bayes	Chi2 score	MinMax	[‘Sex’, ‘Age’, ‘Female Waist Circumference‘(cm), ‘Male Waist Circumference‘(cm), ‘Hypertension categorical‘(1 = normal, 2 = Pre-hypertension, 3 = normal with medicine, 4 = Pre-hypertension with medication, 5 = grade 1, 6 = grade 2), ‘Diabetic‘(1 = taking medicine 2 = not taking medicine), ‘Blood glucose level‘(normal/ prediabetic/ diabetic), ‘Metabolic syndrome‘(yes/no), ‘Total blood pressure‘(yes/no), ‘EPA‘(intake, mg/day)]	0.61	0.56	0.38	0.69	0.70

### LDL category target

As indicated in Table 3, Decision Tree, Random Forest, Naïve Bayes, Neural Network, and Support vector Machine showed a common feature set and KNN showed another one. Neural Network outperformed other algorithms here as well with the accuracy of 0.97, shown in Table 4. Random Forest, KNN, and Decision Tree followed it in terms of accuracy (0.89, 0.87 and 0.82 respectively), however, Decision tree and Random Forest showed slightly better performance for other criteria compared to KNN (1.0 compared to 0.98, 0.93 and 0.97). This problem shows the better ability of these two algorithms in predicting positive and negative samples in different classes. SVM also showed an overall moderate performance with an accuracy of 0.67, precision of 0.78, sensitivity of 0.86 and specificity of 0.89. Naïve Bayse though had some challenges in detecting positive instances for each category (sensitivity of 0.40). Overall, the models demonstrate consistent performance, with some variations noted in accuracy across our dataset and the external validation dataset.

**Table 3:** Feature selection, normalization performance metrics for LDL category target

Algorithm	Feature selection	Normalization	Obtained features from “feature selection” and “normalization” combination	Accuracy	Precision	Sensitivity	Specificity	External dataset accuracy
Decision Tree	Chi2 score	STD	[‘Age’, ‘Level of education‘(illiterate/undergraduate/college), ‘Male Waist Circumference‘(cm), ‘Hypertension categorical(1 = normal, 2 = Pre-hypertension, 3 = normal with medicine, 4 = Pre-hypertension with medication, 5 = grade 1, 6 = grade 2), ‘Hemoglobin’ (g/dl), ‘Metabolic syndrome‘(yes/no), ‘Total blood pressure‘(yes/no), ‘Hypertension grade 2‘(SBP > = 140/DBP > = 90,yes/no), ‘Percent of carbohydrate intake from total daily calories’, ‘Percent of fat intake from total daily calories’]	0.82	1.0	1.0	1.0	0.45
Random Forest	F score	STD		0.89	1.0	1.0	1.0	0.53
Naïve Bayes	Chi2 score	STD		0.72	0.79	0.40	0.82	0.44
**Neural Network**	Chi2 score	STD		**0.97**	1.0	1.0	1.0	**0.71**
Support vector Machine	Chi2 score	STD		0.67	0.78	0.86	0.89	0.52
KNN	F score	Robust	[‘Age’, ‘Level of education‘(illiterate/undergraduate/college), ‘Male Waist Circumference‘(cm), ‘Hypertension categorical‘(1 = normal, 2 = Pre-hypertension, 3 = normal with medicine, 4 = Pre-hypertension with medication, 5 = grade 1, 6 = grade 2), ‘Hemoglobin‘(g/dl), ‘Metabolic syndrome‘(yes/no), ‘Total blood pressure‘(yes/no), ‘Hypertension grade 2‘(SBP > = 140/DBP > = 90,yes/no), ‘Percent of carbohydrate intake from total daily calories’, ‘Percent of fat intake from total daily calories’]	0.87	0.98	0.93	0.97	0.51

**Table 4:** Feature selection, normalization and performance metrics for HDL category target

Algorithm	Feature selection	Normalization	Obtained features from “feature selection” and “normalization” combination	Accuracy	Precision	Sensitivity	Specificity	External dataset accuracy
Decision Tree	Chi2 score	STD	[‘Sex’, ‘Age’, ‘BMI (Body Mass Index) Category’, ‘History of anemia‘(yes/no), ‘Serum Ferritin‘(mcg/l), ‘SerumvitD‘(ng/ml), ‘SerumvitD‘(sufficient/deficient), ‘Metabolic syndrome‘(yes/no), ‘Carbohydrate‘(intake/g/day), ‘VitaminD‘(intake/mcg/day)]	0.65	0.73	0.80	0.68	0.81
**Neural Network**	Chi2 score	STD		**0.99**	0.99	0.99	1.0	0.81
Support vector Machine	fscore	STD		0.64	0.70	0.73	0.67	0.77
KNN	Chi2 score	STD		0.61	1.0	1.0	1.0	**0.86**
Random Forest	Chi2 score	MinMax	[‘Sex’, ‘Age’, ‘History of Heart Disease‘(yes/no), ‘Skipping a meal(yes/no)’, ‘Frequency of eating at home cooked meals’, ‘SerumvitD‘(ng/ml), ‘SerumvitD‘(sufficient/deficient), Metabolic syndrome‘(yes/no), ‘Chromium‘(intake/mcg/day), Iranian oliy bread‘(intake/g/day)]	0.67	1.0	1.0	1.0	0.85
Naïve Bayes	Mutual info	MinMax	[‘Occupation‘(employed or self-employed, student, unemployed), ‘Cigarette smoking(yes/no)’, ‘Skipping a meal‘(yes/no), ‘Serum Ferritin‘(mcg/l), ‘Metabolic syndrome‘(yes/no), ‘Semi-solid oil per capita intake(intake/g/day)’, ‘Saturated fat intake(intake/g/day)’, ‘VitaminB3‘(intake/mg/day), ‘Calcium‘(intake/mg/day), ‘Skinless chicken breast‘(intake/g/day)]	0.65	0.64	0.38	0.81	0.78

### Triglyceride category target

As represented in Table 5, this time only Neural Network reported an individual feature set, and all other algorithms reported another shared one. According to Table 5, we can observe an overall decrement in metric value compared to other targets. The accuracy range was between the minimum of 0.54 (Decision Tree) and the maximum of 0.66 (Neural Network). Both Neural Network and KNN (accuracy of 0.65), showed a reasonable performance in terms of precision, sensitivity, and specificity (0.73, 0.60, 0.88 and 0.93, 0.90, 0.97 respectively). Decision Tree and Random Forest demonstrated high precision and sensitivity (both 0.99), but their low accuracy suggests their inability for identification of classes correctly (0.54 and 0.65). Naïve Bayse and SVM showed moderate performance (accuracies of 0.56 and 0.61), while Naïve Bayse had some challenges with sensitivity (0.50) and SVM demonstrated a little more balanced results for sensitivity and specificity (0.60 and 0.80). In general, the models exhibit stable performance, although slight discrepancies are observed in accuracy between our dataset and the external validation dataset.

**Table 5:** Feature selection, normalization and performance metrics for triglyceride category target

Algorithm	Feature selection	Normalization	Obtained features from “feature selection” and “normalization” combination	Accuracy	Precision	Sensitivity	Specificity	External dataset accuracy
Decision Tree	Chi2 score	STD	[‘Female Waist Circumference‘(cm), ‘FBS‘(mg/dl), ‘Diabetic‘(1 = taking medicine 2 = not taking medicine), ‘Blood glucose level‘(normal/ prediabetic/ diabetic), ‘ALT‘(U/L (units per liter)), ‘Metabolic syndrome‘(yes/no), ‘Manganese‘(intake/mg/day), ‘Fluoride(intake/mg/day)’, ‘Folate‘(intake/mcg/day), ‘Caffeine‘(intake/mg/day)]	0.54	0.99	0.99	0.99	0.52
Random Forest	Chi2 score	STD		0.65	0.99	0.99	0.99	**0.57**
Naïve Bayes	Chi2 score	STD		0.56	0.61	0.50	0.85	0.53
KNN	Chi2 score	STD		0.65	0.93	0.90	0.97	0.52
Support vector Machine	Chi2 score	STD		0.61	0.69	0.60	0.88	0.54
**Neural Network**	Chi2 score	MinMax	[‘Female Waist Circumference‘(cm), ‘Diabetic‘(1 = taking medicine 2 = not taking medicine), ‘Blood glucose level‘(normal/ prediabetic/ diabetic), ‘Metabolic syndrome‘(yes/no), ‘Physical activity‘(1 = inactive, 2 = minimally, 3 = highly active), ‘Manganese‘(intake/mg/day), ‘Fluoride‘(intake/mg/day), ‘Caffeine‘(intake/mg/day), ‘Solid animal oil‘(intake/g/day), ‘Fat tail oil‘(intake/g/day)]	0.66	0.73	0.60	0.88	**0.57**

### HDL category target

Table 4 presents the best combination of feature selection and normalization methods for each algorithm, with Decision Tree, Neural Network, Support Vector Machine, and KNN showing a common feature set. Neural Network achieved the highest accuracy of 0.99, surpassing Random Forest, Decision Tree, and Naïve Bayes. Decision Tree and Random Forest showed superior abilities to detect positive and negative instances across 2 classes (sensitivity 1.0), while Naïve Bayes showed difficulties in detecting positive instances (sensitivity 0.38). The models displayed uniform performance, with minor differences in accuracy between the dataset and the external validation dataset. The Neural Network was the best model for Dyslipidemia Target, achieving an accuracy of 0.97. It showed robust performance in capturing complicated patterns. Decision Tree and Random Forest also demonstrated strong performance in handling complex data relationships. The Neural Network was the best for LDL Category Target, followed by Random Forest and KNN. Decision Tree showed solid performance with an accuracy of 0.82. The Neural Network excelled for HDL Category Target, with an accuracy of 0.99, followed by Random Forest, Decision Tree, and Naïve Bayes.

### Ensembled learning

Table 6 compares ensemble model performance across target variables and machine learning algorithms. Each row represents a specific target variable, and the corresponding ensemble model’s accuracy and F1 score are provided. Ensemble learning methods were applied on algorithms that shared specific normalization and feature selection methods. Ensemble models showed mixed performance compared to individual classifiers across different target variables. While ensemble models for Dyslipidemia and LDL prediction showed higher accuracies, they did not consistently outperform individual classifiers. For instance, in Cholesterol prediction, ensemble models achieved an accuracy of 0.69, comparable to individual classifier accuracies.

**Table 6:** Comparison of ensemble model performance across different target variables and machine learning algorithms

Target	Model	Feature Selection Method	Metric	Ensemble Accuracy	F1 score
Dyslipidemia	Decision Tree	Chi2	MinMax	0.81	1.0
	Random Forest				
	Naïve Bayes				
	Neural Network	F score	STD	0.84	1.0
	Support Vector Machine				
Cholesterol category	Random Forest	Mutual info	STD	0.69	1.0
	KNN				
	Neural Network				
	Support Vector Machine				
LDL category	Decision Tree	Chi2	STD	0.87	1.0
	Naïve Bayes				
	Neural Network				
	Support Vector Machine				
TG category	Decision Tree	Chi2	STD	0.61	1.0
	Naïve Bayes				
	Random Forest				
	Support Vector Machine				
	KNN				
HDL category	Decision Tree	Chi2	STD	0.62	1.0
	Neural Network				
	KNN				

## Discussion

### Addressing research questions

In response to the first research question regarding the performance of different machine learning algorithms, our study demonstrated varying levels of efficacy across the evaluated models. Notably, the Neural Network, particularly the multi-layer perceptron (MLP), consistently outperformed other algorithms in terms of predictive accuracy, precision, recall, and F1 score.

Regarding the second research question on identifying key factors associated with dyslipidemia, our analysis revealed several significant features that were consistently linked to dyslipidemia across different machine learning models.

### Models functioning

The aim of this study was to investigate the application of different machine learning algorithms in predicting the dyslipidemia incidence based on the data of from the LPP Study. However, every method has its own limitations. For instance, the efficacy of using machine learning methods for several domains depends on the feature of the data. The dataset may contain uninterpretable or insignificant values. Therefore, the process of cleaning these ambiguities of the diverse data is a demanding assignment. In addition, choosing a suitable method among these algorithms is challenging due to the different outcomes of methods depending on the data features. Using an incorrect method may lead to confusing results [17].The results of our study demonstrate the potential of machine learning algorithms for the prediction using different feature selection and normalization methods. Among various algorithms evaluated, the Neural Network, specifically multi-layer perceptron (MLP), generally achieved higher results in terms of accuracy, precision recall and F1 score outstanding other common machine learning algorithms. This matter confirms the recent trend in medical predictions, where deep learning algorithms regularly show better potential in understanding the patterns in medical data [43–45].

Furthermore, among other traditional ML models that we used, Random Forest and KNN frequently followed the Neural Network in predictive accuracy. Although among these two, Random Forest also demonstrated marginally better results in other metrics: precision, recall and F1 score. This aligns with the results of previous studies in the field of functional comparison between ML algorithms, where Random Forest regularly outperformed KNN in the context of prediction [46–49]. Generally Random Forest’s reliable superiority in terms of other metrics including precision, recall and F1 score compared to KNN can be related to its multiple abilities such as ensemble learning and noise-resilient approach. In our study, ensemble methods demonstrated diverse performance across different target variables, supporting the results from multiple studies in the field. While ensemble models for Dyslipidemia and LDL prediction showcased higher accuracies, consistent with observations in Gutiérrez-Esparza et al., [41] and Akyea et al., [42] they did not consistently outperform individual classifiers in Cholesterol and TG prediction, echoing results from diverse research such as Buyrukoglu [32] and Tavolinejad [30]. This suggests that the efficacy of ensemble techniques in healthcare prediction tasks is conditional upon factors like dataset characteristics and modeling distinctions.

In Table 7, we present a summary of 2 previous studies that have employed machine learning and deep learning models to predict dyslipidemia incidence. Each row corresponds to a specific study, detailing its methodology, performance metrics, and key findings.

**Table 7:** Summary of previous studies predicting dyslipidemia incidence using machine/deep learning models

Study	Methodology	Performance metrics	Key findings
Marateb et al. (2018)	Supported vector machines, decision trees, multilayer perceptron neural networks, multiple logistic regression	Average accuracy: 92 − 94%	Average precision: 94% Applied for predicting dyslipidemia using gene mutations, family history of diseases, and anthropometric indicators in children and juveniles.
Cui et al. (Year)	Long Short-Term Memory (LSTM)	Accuracy: >95%	LSTM method outperformed traditional recurrent neural network in predicting dyslipidemia in steelworkers.

### Feature selection and clinical relevance

In our study endeavor, we started a thorough assessment focused on dyslipidemia. Throughout this research, we accurately checked five individual target variables directly connected to dyslipidemia. For each of them, we applied a thorough analysis to detect the principal features that showed the strongest associations.

Knowingly, as we examined through the data, we noted a convincing pattern: certain features figured repetitively across all five target variables. This intersection of noteworthy features indicates an important trend well-intentioned of our consideration. Hence, the following part of discussion will evolve around these repeated features, which occur as the keystones joining dyslipidemia related factors. These features, holding the division of being the most regularly recurring among the targets, hold the possibility of unveiling central understandings into the complicated interaction underlying dyslipidemia.

**Fig. 3:**
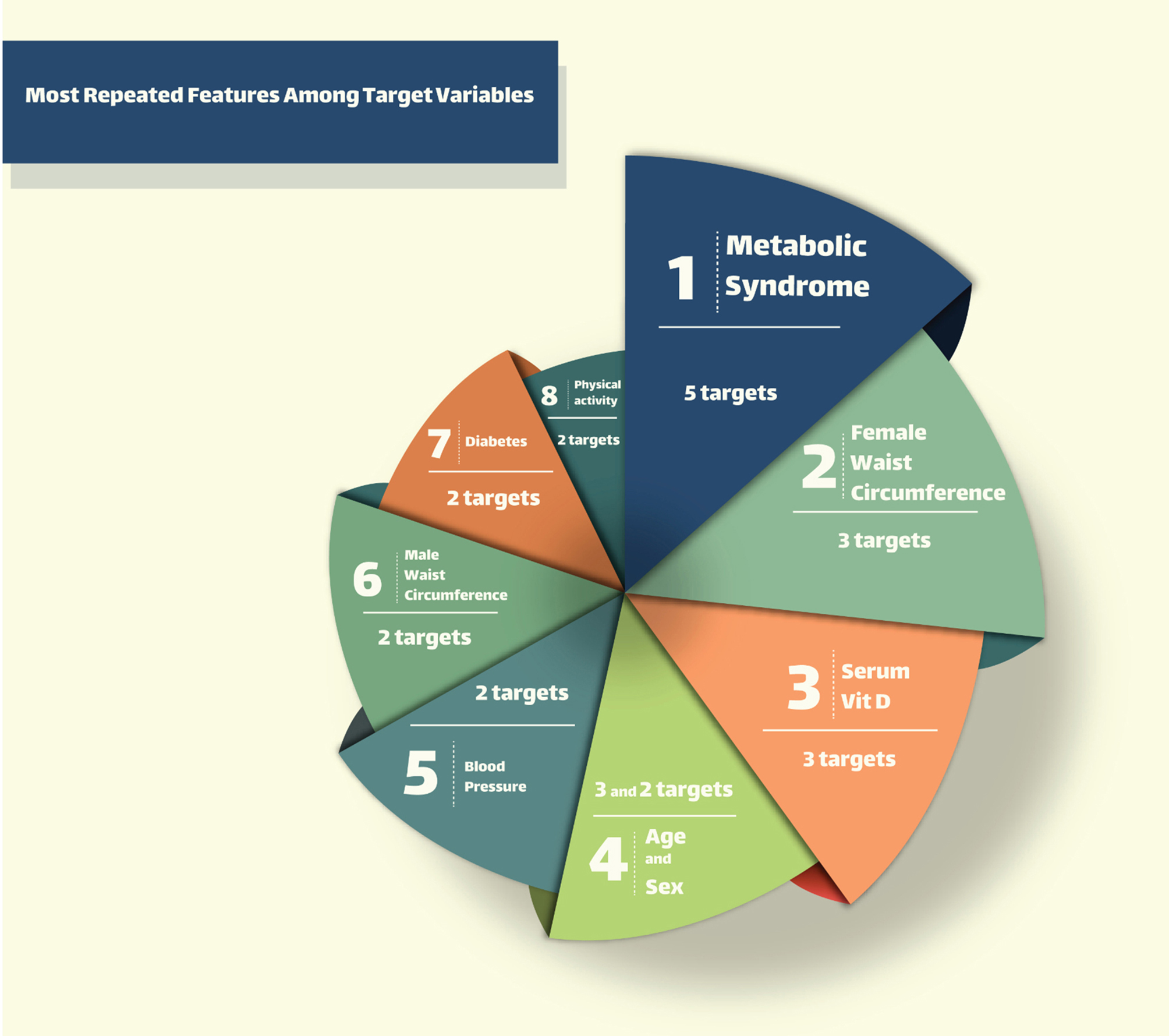
Main dyslipidemia factors: shared features across target variables

In Fig. 3, we provided a visual representation of shared features that highly repeated among the five target variables correlated with dyslipidemia.

#### Metabolic syndrome

According to the NCEP ATP III, the description of metabolic syndrome is related to dyslipidemia, hypertension, and some other features [50]. Standards necessary for its diagnosis include at least five of the following medical situations: belly obesity, high blood pressure, irregular high fasting plasma glucose, raised serum triglycerides and low HDL levels [51]. Therefore, the connection between dyslipidemia and metabolic syndrome is inherent and requires no further clarification.

#### Waist circumference

Our study yielded convincing results suggesting that waist circumference plays a pivotal role in the development of dyslipidemia and its associated markers, which include triglycerides (TG), low-density lipoprotein (LDL), and total cholesterol. These findings held true for both women and men, aligning with existing research that has consistently underscored the relationship between lipid profiles and abdominal fat and obesity. Notably, studies conducted by Ali Chehrei et al. and Mohammed S. Obsa et al., revealed a significant correlation between waist circumference and elevated lipid profiles in Iranian and African populations, respectively [52, 53]. In a study led by B. Longo-Mbenza et al., men with high HDL cholesterol had lower total cholesterol to HDL cholesterol ratios and were less likely to have abdominal obesity [54]. Furthermore, Ren-Nan Feng’s research highlighted waist circumference as a valuable marker within the northern Chinese population [55]. Rodrigo Fernández-Verdejo et al. similarly emphasized that the most common accumulation sequence initiates with abdominal obesity and is followed by dyslipidemia [56].

#### Serum vitamin D

Our discoveries reveal a significant correlation between serum vitamin D levels and dyslipidemia, cholesterol levels, and high-density lipoprotein (HDL) levels. Numerous studies support this obscure relationship between dyslipidemia and vitamin D, indicating their sophisticated interplay. Sever P et al. identified an inverse relationship between plasma vitamin D levels and factors associated with metabolic syndrome, such as cholesterol, low-density lipoprotein, and triglycerides [57]. Furthermore, Conghui Guan et al. study in a Chinese population revealed that vitamin D deficiency and insufficiency were associated with elevated total cholesterol, LDL-C, and triglycerides, along with an increased risk of dyslipidemia [58]. Another investigation by P Karhapää in Finnish men found an inverse association between Serum Vitamin D and total cholesterol, LDL-C, and triglycerides [59]. The connection between vitamin D and lipid profiles is supported by complicated mechanisms. One key mechanism involves the disturbance of lipid metabolism, where insufficient vitamin D levels can distract the balance of lipid synthesis, transport, and utilization, ultimately contributing to elevated lipid levels [60]. Additionally, vitamin D deficiency has been linked to heightened inflammation within the body, exacerbating dyslipidemia by triggering the release of pro-inflammatory molecules that influence lipid regulation [61].

#### Blood pressure

The association between blood pressure and dyslipidemia is a complicated interaction that has been examined across multiple studies. Notably, research investigating the BMI-dyslipidemia interplay in hypertension risk, has underscored the pivotal roles of both overweight and dyslipidemia in elevating the risk [62]. Hanane Ghomari-Boukhatem et al. found a relationship between body mass index (BMI), waist circumference (WC), blood pressure (BP), and dyslipidemia, indicating that overweight (OW) and obese (O) adolescents tend to present these risk factors [63]. Moreover, an examination of blood pressure, Vitamin D deficiency, and dyslipidemia among teenagers uncovered a correlation between these factors, however not all statistically significant [64]. In addition, studies have shed light on the role of ACE enzyme and its correlation with dyslipidemia, implicating this enzyme’s role in early hypertension and dyslipidemia incidence [65]. An inclusive analysis of African population showed a high incidence of dyslipidemia and its impact on hypertension, highlighting the necessity of holistic interventions [66]. Elevated blood pressure boosts atherosclerosis by damaging the endothelium, trapping lipids, and triggering oxidative stress [67]. It also contributes to dyslipidemia through endothelial dysfunction and the influence of hormones like aldosterone [68].

#### Age and sex

Our study reveals persuasive evidence of age and sex disparities within the cholesterol, LDL, and HDL target classes, signifying notable differences in class distribution among patients of varying ages and sexes. The mechanism of aging on dyslipidemia encompasses changes in lipid metabolism. As demonstrated by Humayun A et al., dyslipidemia exhibited an escalating trend with age, both in male and female subjects. In females, dyslipidemia showed a gradual age-related increase across all BMI categories [69]. In alignment with these findings, Cho and colleagues highlighted statistically significant associations between BMI, high blood pressure, and abnormal lipids, with the odds ratios being most prominent in individuals aged 20 to 39, but noticeable trends emerged at older ages [70]. Furthermore, Zhu and associates identified a sex-related difference in the association between dietary cholesterol and dyslipidemia among Chinese metropolitan adults, with sex acting as a significant modifier [71]. Furthermore, comprehensive research conducted at both the national and sub-national levels in Iran has uncovered shifting patterns in plasma cholesterol levels and an increased incidence of total cholesterol [72].

#### Diabetes

According to our results, diabetes appeared to be a significant factor in diverging among different classes in cholesterol and TG targets. Several studies have emphasized on this relationship and other dyslipidemia associated factors. According to Thapa Subarna Dhoj et al., diabetes is related to the high occurrence of dyslipidemia with raised levels of low-density lipoprotein, cholesterol, and triglyceride [73]. Hirano T et al. also demonstrated that serum triglyceride would be main predictor of atherosclerotic cardiovascular disorder in type 2 diabetes [74]. Atherogenic dyslipidemia is also evident in diabetes which includes raised TG-rich lipoproteins, small dense LDL, and low HDL-cholesterol [74]. In type 2 diabetes, metabolic dyslipidemia is illustrated by high triglyceride and low HDL-C, correlated to enhanced cardiovascular risks [75].

#### Physical activity

The interaction between physical activity and dyslipidemia contributors is gradually more obvious, directing to physical activity as a valued factor in dyslipidemia incidence and therefore cardiovascular risk [76]. Research assessing numerous statins in dyslipidemia patients emphasizes physical activity as an applicable modulator of lipid parameters, specifically through physical work’s impact on modifying lipoprotein level and composition [77]. In young individuals, even minimal doses of moderate-to-vigorous physical activity display meaningful lipid profile advances [78]. Participating in as little as 15 to 60 min of moderate to vigorous physical activities daily meaningfully reduces the possibility of high-risk HDL cholesterol and triglyceride values, highlighting the considerable effect of minimal physical activity on cardiovascular health [78].

## Conclusion

The study results underscore the potential of different machine learning algorithms, specifically multi-layer perceptron neural network (MLP), in reaching higher performance metrics such as accuracy, F1 score, sensitivity and specificity, among other machine learning methods. Among other algorithms, Random Forest also showed remarkable accuracies and outperformed K-Nearest Neighbors (KNN) in metrics like precision, recall, and F1 score. The study’s emphasis on feature selection detected meaningful patterns among five target variables related to dyslipidemia, indicating fundamental shared unities among dyslipidemia-related factors. Features such as waist circumference, serum vitamin D, blood pressure, sex, age, diabetes, and physical activity related to dyslipidemia. These results cooperatively highlight the complex nature of dyslipidemia and its connections with numerous factors, strengthening the importance of applying machine learning methods to understand and predict its incidence precisely.

### Benefits and drawbacks of the study

#### Benefits

- Innovative Methodology: Our study establishes the application of several machine learning algorithms, including ensemble methods, for predicting dyslipidemia incidence, using multiple combinations of normalization and feature selection methods to get the most optimized performance.
- High Predictive Accuracy: Utilizing machine learning methods, particularly ensemble methods and Neural Networks, our study consistently achieved high predictive accuracy, precision, recall, and F1 score. This underscores the potential of our developed algorithms in correctly and accurately predicting dyslipidemia incidence, leading to more efficient disorder management approaches.
- Identification of Key Factors: Across broad analysis, our study identified significant features associated with dyslipidemia and other target variables across different machine learning models.

#### Drawbacks

- Data Limitations: While our study benefited from data collected from the “Lifestyle Promotion Project,” accessing additional independent datasets for external validation posed challenges. This limitation restricted our ability to assess the generalizability of our model to diverse populations and settings fully.
- Limitations in External Validation: Regardless of performing external validation, the validation dataset did not support all of our model’s targets. Future research collaborations with other institutions are warranted to address this limitation and enhance the robustness of predictive models.

## Data Availability

Final datasets from the Lifestyle Promotion Project study, are accessible via the GitHub repository linked here : https://github.com/senonaderian/Dyslipidemia.git.
